# What Are the Psychological and Behavioural Outcomes of Vagal Nerve Stimulation and Ketogenic Diet in Children and Young People With Drug-Resistant Epilepsy?

**DOI:** 10.1192/bjo.2024.115

**Published:** 2024-08-01

**Authors:** Oliver Batham

**Affiliations:** South London and Maudsley NHS Foundation Trust, London, United Kingdom

## Abstract

**Aims:**

To systematically review current quantitative evidence for psychological and behavioural outcomes for children with drug-resistant epilepsy being treated with either the ketogenic diet (KD) or vagal nerve stimulation (VNS).

**Methods:**

The review was conducted with a systematic review methodology and the Preferred Reporting Items for Systematic Reviews (PRISMA) tool. The methodology was developed by the author using the PICOS (population, intervention, comparison, outcome, study, design) framework.

Eligibility criteria included children up to 18 years old with epilepsy treated with KD or VNS, and studies which assessed psychological and behavioural outcomes, with validated tools, before and after treatment. Any quantitative design was included. Review articles, meta-analyses, case studies, and case series without a reported mean were excluded. Searches were conducted in four main databases (GlobalHealth, Medline, PsychInfo, Embase) and two grey literature databases (Scopus, Web of Science).

Duplicates were screened using automated processes and then manually. Titles and abstracts were reviewed against eligibility criteria, followed by full texts. Risk of bias was assessed using tools appropriate for the study (the Risk of Bias-2 tool for randomised controlled trials, the JBI checklist for quasi-experimental studies, and the JBI checklist for case series). Included articles were grouped by intervention and by study design for data extraction.

**Results:**

22 studies were identified: 11 for KD, comprising of two randomised controlled trials, one retrospective quasi-experimental study, one retrospective study, two prospective studies, one cross-sectional survey, and four case series; and 11 for VNS, comprising of one randomised controlled trial, two longitudinal observational studies, one prospective observational study, one retrospective study, and six case series.

These studies included a total of 655 participants (523 KD, 132 VNS). There was weak evidence for an improvement in cognitive and behavioural outcomes with both KD and VNS although most studies had methodological weaknesses and were at risk of bias. For both interventions, some studies showed that improvements in outcomes were not related to improvement in seizures, or to reduction in medications.

**Conclusion:**

The evidence base for cognitive and behavioural outcomes following KD or VNS treatment is limited and studies are generally weak and underpowered. Psychological measures used across studies are heterogeneous and difficult to compare. There are little data, but studies raise the possibility that both VNS and KD may affect psychological and behavioural outcomes independently of their effect on seizures. This review supports the need for further research into this area with larger, methodologically robust studies.

